# Bacteria and viruses and clinical outcomes of asthma‐bronchiectasis overlap syndrome: A cohort study

**DOI:** 10.1002/clt2.12331

**Published:** 2024-01-11

**Authors:** Xiao‐xian Zhang, Jia‐hui He, Cui‐xia Pan, Zhen‐feng He, Hui‐min Li, Zhen‐hong Lin, Xiao‐fen Zhang, Lai‐jian Cen, Ri‐lan Zhang, Ming‐xin Shi, Wei‐jie Guan

**Affiliations:** ^1^ State Key Laboratory of Respiratory Disease National Clinical Research Center for Respiratory Disease Guangzhou Institute for Respiratory Health The First Affiliated Hospital of Guangzhou Medical University Guangzhou Guangdong China

**Keywords:** asthma, asthma‐bronchiectasis overlap syndrome, atopy, exacerbation

## Abstract

**Background:**

Despite the high prevalence of co‐existing bronchiectasis and asthma (asthma‐bronchiectasis overlap syndrome [ABOS]), little is known regarding the dominant pathogens and clinical correlates.

**Objective:**

To investigate the bacteria and viruses which differentially dominate in ABOS (including its subtypes) compared with bronchiectasis alone, and determine their relevance with bronchiectasis severity and exacerbations.

**Methods:**

This was a prospective observational cohort study conducted between March 2017 and August 2023. We included 81 patients with ABOS and 107 patients with bronchiectasis alone. At steady‐state baseline, patients underwent comprehensive assessments and sputum collection for bacterial culture and viral detection (quantitative polymerase‐chain‐reaction). Patients were followed‐up to record exacerbation and spirometry.

**Results:**

Patients with ABOS had significantly higher symptom burden and exacerbation frequency than those with bronchiectasis alone. Despite similar pathogen spectrum, the rate of bacteria–virus co‐detection increased less substantially at acute exacerbations (AE) onset than at steady‐state compared with bronchiectasis alone. Pathogenic bacteria (particularly *Pseudomonas aeruginosa*) were detected fairly common (exceeding 50%) in ABOS and were associated with greater severity of bronchiectasis when stable and conferred greater exacerbation risks at follow‐up. Viral but not bacterial compositions changed substantially at AE onset compared with clinical stability. Higher blood eosinophil count, moderate‐to‐severe bronchiectasis and non‐atopy were associated with higher odds of bacterial, but not viral, detection (all *p* < 0.05).

**Conclusion:**

Detection of bacteria or virus is associated with bronchiectasis severity or clinical outcomes in ABOS. This highlights the importance of integrating sputum microbial assessment for ascertaining the dominant pathophysiology (atopy vs. infection) and longitudinal trajectory prediction in ABOS.

## 1 INTRODUCTION

Both asthma and bronchiectasis are common chronic respiratory diseases (CRDs).^1^ For bronchiectasis, the pathological bronchial dilation primarily stems from chronic airway infection and inflammation—the latter has been the shared pathophysiological component of asthma.^2^ Depending on the clinical milieu, asthma may develop among some patients with bronchiectasis, while bronchiectasis might develop among some asthmatic patients through the trajectory of progression.^3^ Bronchiectasis is common among patients with severe asthma. For instance, in a systematic review, the prevalence of asthma in patients with bronchiectasis ranged between 15% and 30.2%. Patients with co‐existing bronchiectasis and asthma (asthma‐bronchiectasis overlap syndrome [ABOS]) reportedly had a higher exacerbation frequency than those with bronchiectasis alone.^1^, ^4^ Despite the high prevalence, little is known regarding the pathophysiology of ABOS. Moreover, there is no study that explores the causal relationship between asthma and bronchiectasis.

Chronically inflamed airways have formed a niche for infection with respiratory pathogens. An outgrowth of pathogenic bacteria (e.g., *Haemophilus influenzae* [HI]) has been implicated in asthma^5^ and bronchiectasis^6^ and correlates significantly with the disease severity, inflammatory responses and prognosis.^6^ Apart from bacteria, some viruses have been linked to heightened inflammation and exacerbation of asthma^7^, ^8^ and bronchiectasis.^9^

Accumulating evidence has begun to unravel the interactions between bacteria and viruses in CRDs. Rhinovirus detection frequently coincided with bacterial detection, and rhinovirus co‐existing with *Moraxella catarrhalis* or *Streptococcus pneumoniae* has been associated with greater symptom burden and exacerbation risks in asthmatic children.^10^ Furthermore, viral detection, isolation of new bacteria, and bacterial isolation plus viral detection have been consistently associated with bronchiectasis exacerbations.^11^

We hypothesized that some pathogens differentially dominate in ABOS (including its subtypes) compared with bronchiectasis alone. Here, we elucidated the spectra of bacteria and viruses at clinical stability and exacerbations of ABOS, and their clinical correlates. These may help better appraise the role of bacterial detection and the clinical relevance in ABOS.

## 2 METHODS

### 2.1 Study participants

We consecutively recruited adults from out‐patient clinics between March 2017 and August 2023. Clinically significant bronchiectasis was diagnosed as high‐resolution computed tomography (HRCT) manifestations (an inner airway‐artery diameter ratio of 1.5 or more, an outer airway‐artery diameter ratio of 1.5 or more, a lack of tapering of the airways, and visibility of airways in the periphery) compatible with respiratory symptoms (particularly daily cough, chronic mucopurulent or purulent sputum, a history of exacerbations).^12^ Eligible patients with ABOS should be in steady‐state at baseline ‐ remaining exacerbation‐free and had no antibiotics used (except for low‐dose macrolides) for >4 weeks. Acute exacerbations (AEs) denoted a significant deterioration of three or more symptoms persisting for at least 48 h that required immediate changes in treatment, according to *European Respiratory Society* expert consensus.^13^ Asthma was a physician‐diagnosis based on *Global Initiative for Asthma* guidelines—respiratory symptoms (wheezing, shortness of breath, cough or chest tightness) plus variable expiratory airflow limitation based on the significant bronchodilator response or airway hyperresponsiveness.^1^ Severe asthma was defined as asthma requiring level 4/5 treatments to maintain asthma control, or asthma that remained uncontrolled despite the above‐mentioned treatments. ABOS was diagnosed among patients with co‐existing asthma and bronchiectasis. ABOS was further stratified by the presence (severe asthma‐ABOS [SA‐ABOS]) or absence (non‐severe asthma [(NSA‐ABOS]) of severe asthma.

The key exclusion criteria were active tuberculosis, malignancy, eosinophilic granulomatosis with polyangiitis, allergic bronchopulmonary aspergillosis, traction bronchiectasis, insufficient sputum yield, and pregnancy or lactation.

Ethics approval was obtained from the Ethics Committee of The First Affiliated Hospital of Guangzhou Medical University (Medical Ethics [2012] the 33^th^; Medical Ethics [2020] the 156^th^). All patients signed written informed consent.

### 2.2 Study design

This study was divided into two sections. Section 1 was a prospective observational cohort study (May 2018 to August 2023) investigating bacterial and viral detection in steady‐state ABOS and the clinical correlates. Section 2 was an extended study which added historical sputum samples collected since March 2017 (with paired bacterial and viral detection data),^14^ which analyzed bacterial and viral detection at AE onset of ABOS. Records of AE onset were incomplete between March 2017 and April 2018, because some patients were lost to follow‐up. Therefore, the association between bacterial detection and future risk of AE was analyzed in Section 1 only.

### 2.3 Procedures

At initial visits, we collected clinical information, and performed etiological work‐up, spirometry,^15^ chest HRCT,^16^ fractional exhaled nitric oxide assay (if indicated) and symptom questionnaire which inquired upper and lower airway symptoms (rating the severity with visual analog scale (VAS, range: 0–10]). We rated bronchiectasis severity with Bronchiectasis Severity Index (BSI)^17^, ^18^ and E‐FACED score.^19^ We ascertained atopic status according to the total or specific immunoglobulin *E* titers for allergens (including inhaled allergens, molds, and food proteins) in serum or skin‐prick test findings with standard array testing. Blood eosinophil count was stratified into high (>300/μL) or low eosinophil (≤300/μL) subgroups.^1^

Spontaneous sputum collection (aided with chest physiotherapy) was prioritized, or alternatively, induced sputum with 3% saline if sputum yield was insufficient. We performed a quality check and split sputum for differential cell counts, bacterial culture and viral detection with quantitative polymerase‐chain‐reaction. We defined repeated detection of the same pathogenic bacteria as the isolation of the same bacteria at least twice within a year, at intervals of 3 months or more.

Patients immediately contacted investigators upon significant symptom aggravation. We followed patients via telephone every 2 months and scheduled outpatient clinics every 3–6 months for recording exacerbation history and spirometry. Sputum samples were collected prior to the prescription of antibiotics, and AE samples were collected on the first day of AE.

### 2.4 Pathogen detection

Laboratory techniques for pathogen detection included bacterial culture and viral detection. We conducted bacterial culture by homogenizing fresh sputum.^20^ Pathogenic bacteria included, but not limited to, *Pseudomonas aeruginosa* (PA), HI, *Haemophilus parainfluenzae*, *Klebsiella pneumoniae*, *Streptococcus pneumoniae*, *Streptococcus aureus* and *Escherichia coli*. We extracted viral nucleic acids using extraction kit (TaKaRa MiniBEST Viral RNA/DNA Extraction Kit Ver. 5.0) and used TaqMan reverse‐transcriptase PCR to detect 16 common respiratory viruses^9^, ^14^: rhinovirus, influenza virus A/B, parainfluenza virus1‐4, human coronavirus (HCoV‐229E, OC43, NL63 and HKU1), respiratory syncytial virus, adenovirus, enterovirus, bocavirus and human metapneumovirus. A cycle threshold <40 was deemed positive for respiratory viruses.

### 2.5 Statistical analysis

Sample size was calculated using PASS software version 11.0.7. The proportion of bronchiectasis patients with bacterial isolation and viral detection was 59.8% and 11.4% at stable visits, and 61.9% and 29.0% at AEs, respectively.^11^ Therefore, we estimated to recruit 102 ABOS patients with two‐sided significance of 0.05 and power of 80%, assuming 20% dropout at follow‐up.

Data were presented as mean ± standard deviation (SD), median (interquartile range [IQR]) for continuous variables, and counts (proportion) for categorical variables. We explored the association between pathogen detection and the odds of AEs compared with stable visits by using generalized estimating equations, with a logit link and repeated observations among study participants. We analyzed continuous variables with *t*‐test, analysis‐of‐variance, Mann‐Whitney, or Kruskal‐Wallis test, depending on the variable distribution, and compared categorical variables with Chi‐square or Fisher exact test. Univariate Logistic analysis was conducted to explore the factors of positive sputum culture or repeated detection of the same pathogenic bacteria, and detection or repeated detection of PA, with results being reported as odds ratio (OR) and 95% confidence interval (95% CI). We employed multiple Logistic regression models to assess confounders. Data with *p* ≤ 0.10 were entered into multivariate analysis as covariates using the backward selection technique. The variables adjusted consisted of the age, 24‐h sputum, allergic rhinitis, Reiff score, FEV_1_ pred%, FEV_1_/FVC, the number of bronchiectatic lobes, blood neutrophil count, blood eosinophil count, and sputum neutrophil count (percentage). Missing data were not imputed. The future risk of AE was analyzed with Kaplan‐Meier model and compared with the log‐rank test. SPSS (version 23.0) and Graphpad Prism (version 5.0) were used for statistical analyses. *p* < 0.05 was considered statistically significant.

## 3 RESULTS

### 3.1 Section 1

#### 3.1.1 Baseline characteristics associated with pathogen detection in ABOS

**FIGURE 1:**
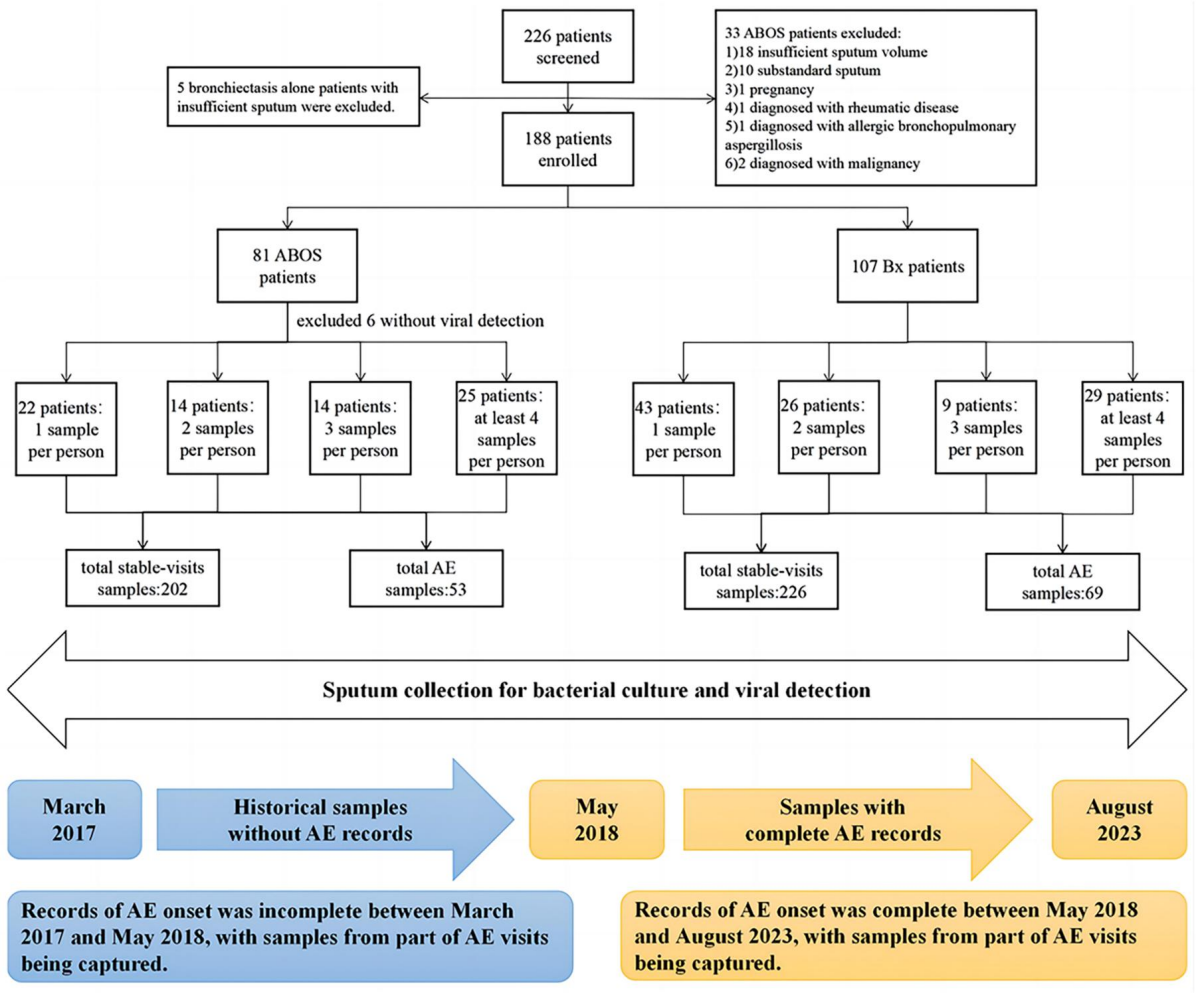
Flow chart of patient recruitment. ABOS, asthma‐bronchiectasis overlap syndrome; AE, acute exacerbations of bronchiectasis; Bx, bronchiectasis alone.

Of 114 patients with ABOS screened, 81 were enrolled. The reasons of exclusion are demonstrated in Figure 1. Eighty one Patients with ABOS were divided into bacterial culture positive and negative groups, or PA group and non‐PA group. After excluding 10 patients with missing viral detection status at baseline, 75 patients with ABOS were stratified into viral positive and negative groups. Bacterial detection was associated with a longer disease duration, higher daily sputum yield, higher BSI and the modified Reiff scores, more severe airflow limitation, and a more frequent use of muscarinic antagonists. Sputum culture negative was more likely to be associated with previous wheezing episodes, allergic rhinitis, and blood eosinophilia (Table 1). The proportion of patients with allergy history (e.g. allergic rhinitis) was significantly higher in the PA‐negative group than in the PA‐positive group. At baseline, ABOS patients with PA or any bacteria detected had more severe bronchiectasis. However, the clinical characteristics did not differ significantly between the viral positive and negative groups at baseline (Table S1).

**TABLE 1:** Baseline clinical characteristics in ABOS patients with or without bacterial detection when clinically stable.

	Positive sputum culture (*n* = 45)	Negative sputum culture (*n* = 36)	*p* Value	PA positive (*n* = 31)	PA negative (*n* = 50)	*p* Value
Age (yrs)	47.8 ± 13.6	49.6 ± 12.2	0.539	50.3 ± 11.9	47.5 ± 13.5	0.340
Females, *n* (%)	29 (64.4%)	25 (69.4%)	0.635	19 (61.3%)	35 (70.0%)	0.419
Body‐mass index (kg/m^2^)	21.0 (5.2)	21.6 (3.3)	0.846	22.1 (4.8)	21.3 (4.0)	0.767
Duration of symptom onset (yrs)	20.0 (17.5)	10.0 (13.3)	**<0.001**	22.0 (19.0)	13.0 (14.0)	**<0.001**
Wheeze, *n* (%)	14 (31.1%)	19 (52.7%)	**0.049**	10 (33.2%)	23 (46.0%)	0.221
24‐h sputum ≥10 mL, *n* (%)	32 (71.1%)	16 (44.4%)	**0.015**	24 (77.4%)	24 (48.0%)	**0.009**
Previous smoker, *n* (%)	1 (2.2%)	6 (16.7%)	**0.041**	1 (3.2%)	6 (12.0%)	0.238
Family history of allergic disease, *n* (%)	16 (35.5%)	19 (52.7%)	0.120	9 (29.0%)	26 (52.0%)	**0.043**
Rhinitis or sinusitis, *n* (%)	28 (62.2%)	28 (77.7%)	0.132	19 (61.3%)	37 (74.0%)	0.229
Allergic rhinitis, *n* (%)	16 (35.5%)	24 (66.7%)	**0.005**	11 (35.5%)	29 (58.0%)	**0.049**
Etiology						
Post‐infective, *n* (%)	9 (20.0%)	9 (25.0%)	**0.591**	6 (19.4%)	12 (24.0%)	**0.625**
Idiopathic, *n* (%)	19 (42.2%)	19 (52.8%)	**0.344**	21 (67.7%)	24 (40.8%)	**0.082**
Post‐tuberculous, *n* (%)	2 (4.4%)	4 (11.1%)	**0.399**	2 (6.5%)	4 (8.0%)	＞**0.999**
Primary immunodeficiency, *n* (%)	1 (2.2%)	1 (2.8%)	＞**0.999**	0 (0.0%)	2 (4.0%)	**0.522**
Others, *n* (%)	7 (15.6%)	3 (8.3%)	**0.499**	2 (6.5%)	8 (16.0%)	**0.303**
Influenza vaccination within 1 year, *n* (%)	5 (11.1%)	3 (8.3%)	0.677	5 (16.1%)	3 (6.0%)	0.249
Pneumococcal vaccination within 5 years, *n* (%)	4 (8.9%)	6 (16.7%)	0.290	3 (9.6%)	7 (14.0%)	0.734
Exacerbations frequency in the previous year	1.0 (2.0)	1.0 (1.0)	0.928	1.0 (2.0)	1.0 (1.3)	0.438
No. of patients hospitalized in the previous year	0.0 (0.5)	0.0 (0.0)	0.268	0.0 (1.0)	0.0 (0.0)	0.105
Severe asthma, *n* (%)	6 (13.3%)	9 (25.0%)	0.179	5 (16.1%)	10 (20%)	0.663
Bronchiectasis severity index	7.0 (5.0)	3.5 (3.5)	**<0.001**	8.0 (5.0)	4.0 (3.0)	**<0.001**
E‐FACED score^b^	3.0 (2.0)	1.0 (2.8)	**<0.001**	4.0 (2.0)	1.0 (2.3)	**<0.001**
HRCT Reiff score^c^	9.3 ± 4.2	6.8 ± 4.0	**0.009**	8.0 (7.0)	7.0 (5.0)	**0.004**
No. of bronchiectatic lobes^d^	5.0 (2.0)	3.0 (2.0)	**0.011**	5.0 (3.0)	4.0 (3.0)	**0.011**
FEV_1_ (%)	51.9 ± 14.9	65.9 ± 19.4	**<0.001**	50.6 ± 15.6	62.8 ± 18.5	**0.003**
FEV_1_/FVC (%)	58.5 ± 11.5	64.4 ± 13.3	**0.036**	57.0 ± 12.4	63.7 ± 12.1	**0.019**
Laboratory test findings						
White blood cells (*10^9^/L)	7.2 (2.4)	6.8 (2.7)	0.314	7.6 ± 2.0	7.1 ± 1.9	0.219
Blood neutrophils (%)	63.6 ± 12.5	60.5 ± 12.9	0.136	64.6 ± 7.6	60.8 ± 9.9	0.067
Blood eosinophils (%)	1.7 (1.8)	3.0 (3.0)	**0.003**	1.9 (1.9)	2.5 (2.7)	0.194
C‐reactive protein^a^ (mg/dL)	0.4 (0.5)	0.2 (0.4)	0.063	0.4 (0.4)	0.2 (0.5)	**0.030**
Total immunoglobulin E^a^ (KU/ml)	39.8 (114.3)	94.4 (172.4)	0.075	41.7 (153.3)	78.8 (162.4)	0.517
Fractional exhaled nitric oxide^a^; (ppb)	14.0 (16.0)	16.0 (23.0)	0.508	14.0 (15.5)	15.0 (17.5)	0.456
Sputum neutrophils^a^ (%)	96.2 (3.8)	92.3 (13.2)	**0.007**	96.5 (2.8)	93.4 (9.4)	**0.003**
Sputum eosinophils^a^ (%)	1.0 (1.7)	1.2 (2.8)	0.116	1.1 (1.5)	1.2 (2.5)	0.967
Medications						
Low‐dose macrolides, *n* (%)	5 (11.1%)	6 (16.7%)	0.468	4 (12.9%)	7 (14.4%)	1.000
Inhaled corticosteroids, *n* (%)	14 (31.1%)	16 (44.4%)	0.217	10 (32.3%)	20 (40.0%)	0.483
Oral corticosteroids, *n* (%)	0 (0.0%)	4 (11.1%)	**0.035**	0 (0.0%)	4 (8.0%)	0.292
Long‐acting muscarinic antagonists, *n* (%)	25 (55.6%)	11 (30.6%)	**0.024**	19 (61.3%)	17 (34.0%)	**0.016**
Long‐acting beta‐agonists, *n* (%)	20 (44.4%)	17 (47.2%)	0.803	15 (48.4%)	22 (44.0%)	0.700

Abbreviations: FEV_1_, forced expiratory volume in one second; FVC, forced vital capacity.

^a^ Seventy eight patients had undergone sputum cytology examination, 74 patients had undergone total immunoglobulin E testing, 74 patients had undergone fractional exhaled nitric oxide testing, 77 patients had undergone C‐reactive protein testing.

^b^ E‐FACED score: an integrated clinical severity metric.

^c^ HRCT Reiff score: a metric reflecting the radiological severity of bronchiectasis.

^d^ No. of bronchiectatic lobes: a metric reflecting the radiological extension of bronchiectasis.

Bold font: *p* < 0.05, statistically significant differences.

#### 3.1.2 Risk factors for bacterial detection at baseline

In light of the differential bacterial spectrum between asthma and bronchiectasis, we interrogated the factors predicting the detection of any pathogenic bacteria at baseline when clinically stable among patients with ABOS. To this end, we probed the association between different clinical variables and the detection of pathogenic bacteria using a multivariable logistic regression model. Because the BSI and E‐FACED scores included repeated detection of any pathogenic bacteria or PA, we used the modified Reiff score and FEV_1_ pred% to analyze the risk factors for bacterial detection at baseline. In the multivariable logistic regression model, higher FEV_1_ pred% (OR: 0.95, 95% CI: 0.91–0.99, *p* = 0.010) was associated with no bacterial detection. A longer duration of symptom onset (OR: 1.06, 95% CI: 1.02–1.11, *p* = 0.006) was significantly associated with PA detection in ABOS. A longer duration of symptom onset (OR: 1.06, 95% CI: 1.01–1.11, *p* = 0.009) and sputum neutrophilia (OR: 1.14, 95% CI: 1.01–1.29, *p* = 0.031) were significantly associated with the repeated PA detection when clinically stable (Table 2).

**TABLE 2:** Risk factors for bacterial detection or repeated detection in ABOS at baseline.

	Risk factors	Univariate analysis OR (95% CI)	*p*	Multivariate analysis OR (95% CI)	*p*
Positive bacterial cultures	FEV_1_ pred%^a^	0.95 (0.93–0.98)	0.001	0.95 (0.91–0.99)	0.010
Repeated detection of the same bacteria	FEV_1_ pred%^b^	0.98 (0.95–1.00)	0.070	0.96 (0.92–0.99)	0.016
	Blood eosinophils (%)^b^	0.73 (0.56–0.97)	0.027	0.67 (0.49–0.92)	0.011
*Pseudomonas aeruginosa* detection	The duration of symptom onset	1.06 (1.02–1.10)	0.002	1.06 (1.02–1.11)	0.006
Repeated detection of *Pseudomonas aeruginosa*	The duration of symptom onset^c^	1.06 (1.02–1.11)	0.004	1.06 (1.01–1.11)	0.009
	Sputum neutrophils # (%)^c^	1.13 (1.00–1.28)	0.048	1.14 (1.01–1.29)	0.031

*Note*: Also adjusted: the duration of symptom onset^a,b,c^, wheezing^a^, atopy^a^, ex‐smoker^a^, sputum eosinophils(%)^a^, C‐reactive protein^b^, No. Of patients hospitalized in the previous year^b^.

Abbreviation: FEV1 pred%, the forced expiratory volume in one second percentage predicted.

#### 3.1.3 Baseline pathogen detection and longitudinal clinical outcomes

**FIGURE 2:**
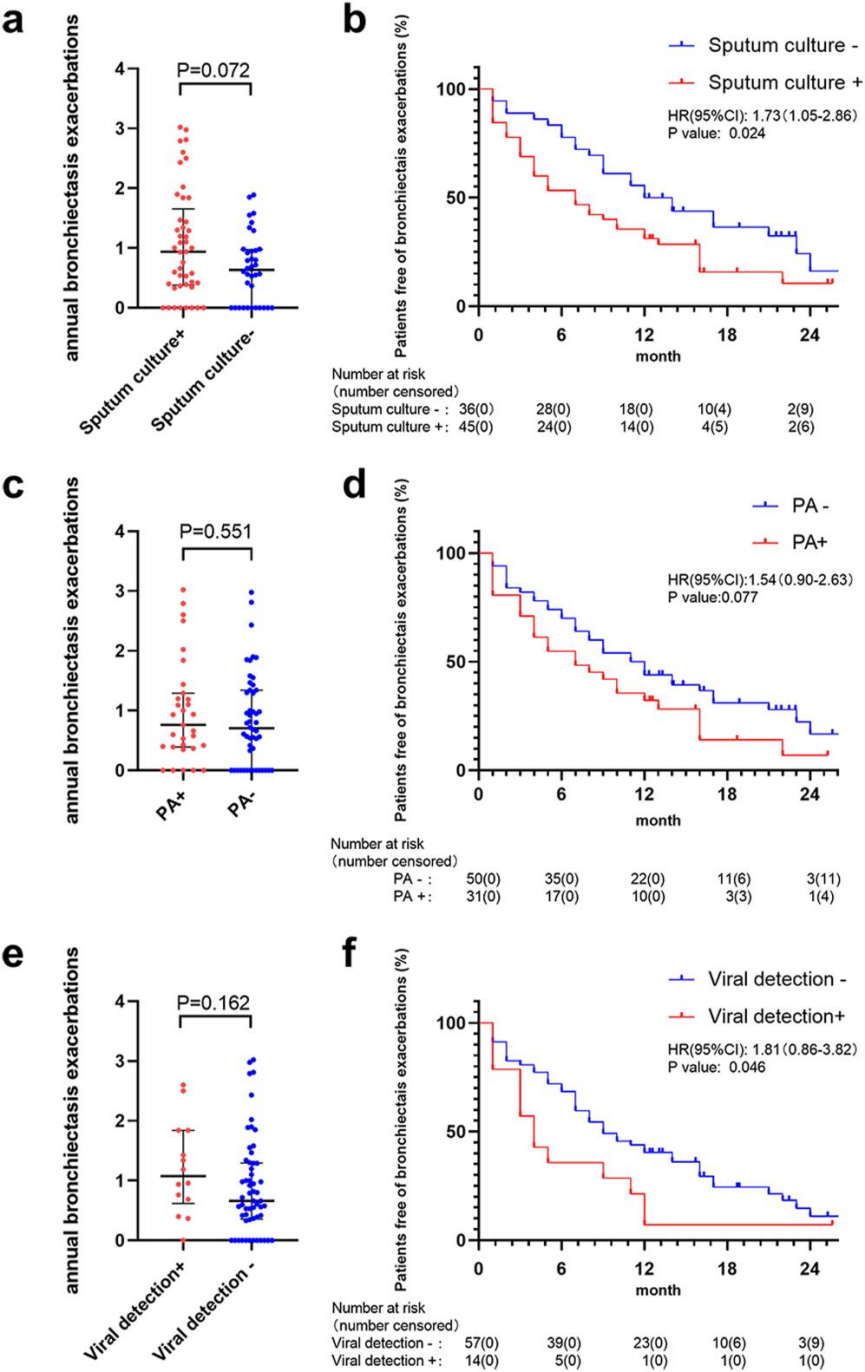
The risk of bronchiectasis exacerbations between ABOS patients with or without pathogen detection during the 2‐year longitudinal follow‐up. Annual bronchiectasis exacerbation frequency (a,c,e). Proportion of patients free from bronchiectasis exacerbation (b,d,f). PA, *Pseudomonas aeruginosa*.

We next explored whether pathogen detection at baseline could predict the clinical outcomes during longitudinal follow‐up. During the median follow‐up of 25.3 (IQR:14.2) months, 81 patients with ABOS reported 137 AE episodes (17 requiring hospitalization). 53 patients had paired spirometric reassessment. The forced expiratory volume in one second percentage predicted (FEV_1_ pred%) improved considerably from baseline to follow‐up (mean: 56.4% vs. 62.0%, *p* < 0.001), which did not differ significantly when stratified by bacterial (including PA and virus) detection status (*p* > 0.05) (Figure S1). At baseline, the annualized frequency of AE did not differ significantly when stratified by bacterial (including PA and virus) detection status (*p* > 0.05). Patients with bacterial detection at baseline had a significantly shorter time to the first AE than those without (median: 7 vs. 13 months, HR: 1.73, 95% CI: 1.05–2.86). Patients with viral detection at baseline also had a significantly shorter time to the first AE than those without (median: 4 vs. 9 months, HR: 1.81, 95% CI: 0.86–3.82). However, no significant difference in this metric was identified between the PA and non‐PA groups (Figure 2).

### 3.2 Section 2

#### 3.2.1 Patient recruitment

To investigate the characteristics of bacterial plus viral detection in ABOS, we excluded six patients with missing viral detection status, leaving 75 patients in the final analysis. One hundred and eleven historical samples from patients with ABOS (March 2017 to May 2018) were incorporated. Of 112 patients with bronchiectasis alone, five were excluded because of insufficient sputum volume, with 107 being included in the final analysis.

The median follow‐up was 25.3 (IQR:14.2) months. Seventy five patients with ABOS provided 202 sputum samples from steady‐state visits and 53 from AE visits (83 historical samples from steady‐state visits and 28 from AE visits). Each patient provided a median of 3.4 samples. Among patients who provided multiple samples, the median interval from the earliest to the latest sampling was 29.2 (IQR: 12.8) months. The 75 patients reported 136 AEs during this time interval (53 AEs with hospital visits). The 107 patients with bronchiectasis alone provided 226 sputum samples from steady‐state visits and 69 from AE visits (median: two samples per patient). Patient recruitment is shown in Figure 1.

Patient characteristics are shown in Table S2–4. Compared with patients with bronchiectasis alone, those with ABOS had a higher body‐mass index, more frequently received influenza or Pneumococcal vaccination and inhaled medications, and had higher prior exacerbation frequency and more severe airflow limitation. At baseline, the clinical characteristics did not differ significantly between the whole and the AE cohort among patients with ABOS and bronchiectasis alone.

#### 3.2.2 Bacterial and viral spectrum of ABOS compared with bronchiectasis alone

**FIGURE 3:**
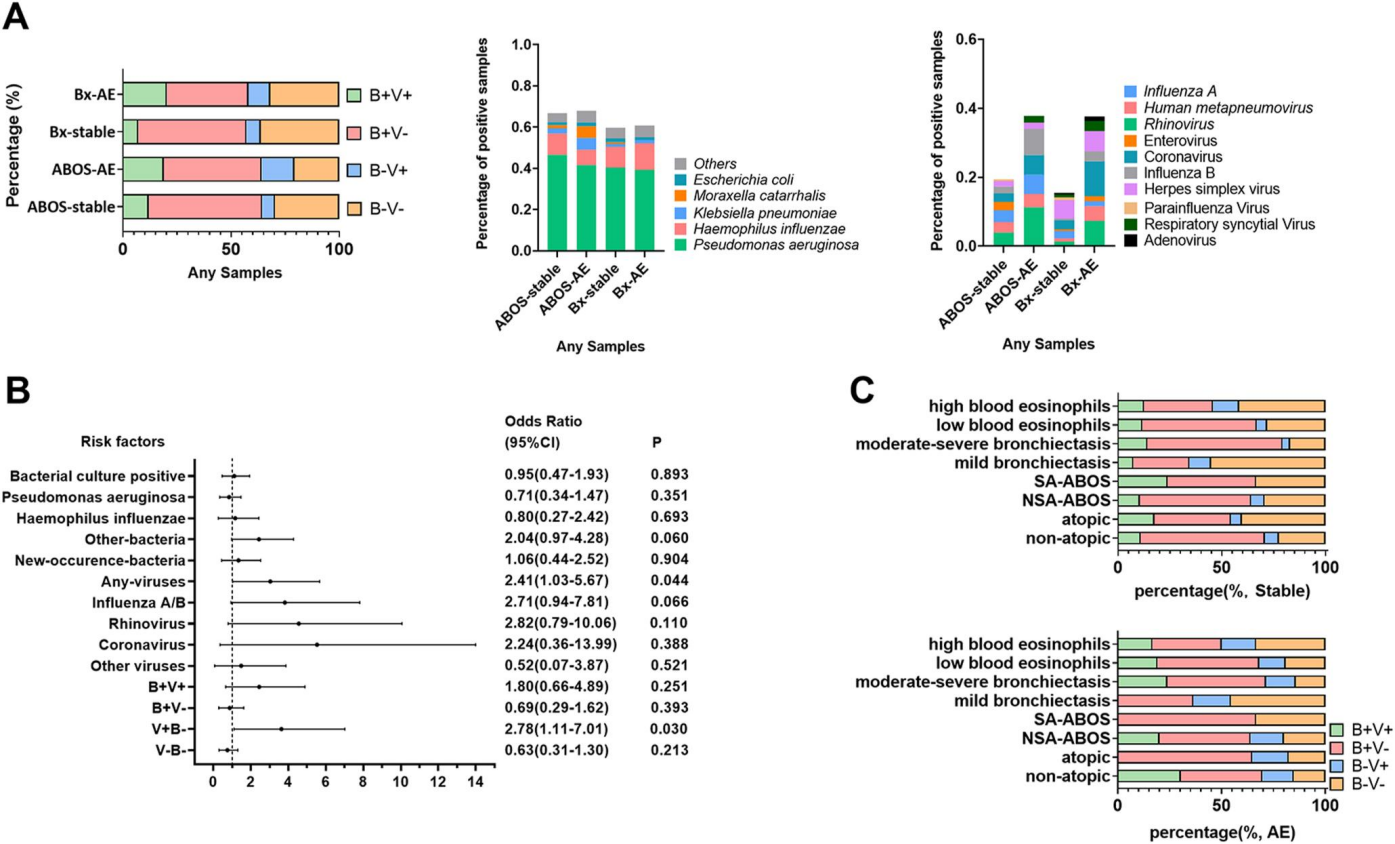
The detection rate and composition of bacteria and viruses at steady‐state and exacerbation onset of ABOS. ABOS, asthma‐bronchiectasis overlap syndrome; AE, acute exacerbations of bronchiectasis; B+V+, both bacteria and viruses detected; B+V−, any pathogenic bacteria detected but no viruses detected; B−V+, viruses detected but no pathogenic bacteria detected; B−V−, no bacteria and viruses detected; Bx, bronchiectasis; NSA‐ABOS, ABOS with non‐severe asthma; SA‐ABOS, ABOS with severe asthma. New‐occurrence‐bacteria denotes sputum culture switching from negative to positive, or from one pathogenic bacterium to other pathogenic bacterium.

We next analyzed the pathogen spectrum of ABOS by comparing it with bronchiectasis alone. First, we focused on the bacterial detection rate and the bacterial spectrum. The detection rate of bacteria alone (45.3% vs. 52.5%) or in combination with virus (18.9% vs. 11.9%) did not increase significantly at AE onset compared with steady‐state (both *p* > 0.05) (Figure 3). The most common bacteria were PA in steady‐state and AE visits (46.5% vs. 41.5%), followed by HI (10.4% vs. 7.5%). Despite a higher detection rate of *Klebsiella pneumoniae*, *Moraxella catarrhalis* and *Escherichia coli* at AEs, bacterial detection was not associated with AE onset in ABOS. Overall, these findings mirrored those among patients with bronchiectasis alone, except that the rate of bacteria plus virus co‐detection increased substantially at AE onset than at steady‐state (20.3% vs. 7.1%, *p* = 0.003) in bronchiectasis alone.

Next, we probed the detection rate and spectrum of viruses in ABOS. At AE onset, patients with ABOS yielded markedly higher detection rates of viruses, but not bacteria, than at steady‐state (15.1% vs. 5.9%, *p* = 0.027). Viral detection occurred more frequently during AEs than during steady‐state (OR: 2.41, 95%CI: 1.03–5.67). The dominant viruses were rhinovirus (4.0% vs. 11.3%) and influenza A (3.5% vs. 5.7%) at steady‐state and AE onset. Other common viruses consisted of human metapneumovirus, influenza B virus and coronavirus. Despite similar trends of viral detection rate at AE compared with steady‐state, the most common viruses detected in patients with bronchiectasis alone were herpes simplex virus, coronavirus and rhinovirus. In the univariate logistic regression model, pathogen detection did not differ significantly between patients with ABOS and bronchiectasis alone (Table 3).

**TABLE 3:** Risk factors for pathogen detection at baseline visit and acute exacerbations of bronchiectasis visits among patients with ABOS.

Patients with ABOS	Factors	Univariate analysis OR (95% CI)	*p*
Baseline visits	B+V+	1.77 (0.91–3.44)	0.092
	B−V+	0.89 (0.41–1.95)	0.767
	B+V−	1.10 (0.76–1.61)	0.609
	B−V−	0.74 (0.50–1.11)	0.149
Exacerbation visits	B+V+	0.91 (0.37–2.26)	0.845
	B−V+	1.58 (0.53–4.66)	0.412
	B+V−	1.37 (0.66–2.83)	0.398
	B−V−	0.56 (0.24–1.29)	0.173

Abbreviations: B+V+, both bacteria and viruses detected; B+V−, any pathogenic bacteria detected but no viruses detected; B−V+, viruses detected but no pathogenic bacteria detected; B−V−, no bacteria and viruses detected.

#### 3.2.3 Clinical characteristics differentiating AEs with different pathogens

Having demonstrated the symptoms and laboratory test findings that differentiated bacterial from viral detection in our previous study, we then interrogated whether similar conclusions would apply to ABOS. According to the virus detection status at AEs (Figure 3), we classified patients with ABOS into virus‐positive (*n* = 11) versus virus‐negative (*n* = 27) strata, or into viral detection without bacterial detection (V+B−, *n* = 6) versus other pathogen strata (*n* = 32). At AEs, neither upper nor lower airway symptoms differed when comparing these two strata (Table S5). Symptom questionnaires were obtained from 38 (71.7%) AEs, blood routine test from 27 (50.9%) AEs, and C‐reactive protein levels from 26 (49.1%) AEs. Again, none of these metrics differed significantly between these two strata (Figure S2 and S3). However, compared with other pathogen strata, patients in viral detection without bacterial detection strata more frequently reported cough, breathlessness and wheeze at AE onset.

#### 3.2.4 Subgroup analysis of pathogen detection at steady‐state

Finally, we compared the pathogen detection rate across different strata, particularly the blood eosinophil count and bronchiectasis severity—the core metrics determining the heterogeneity of ABOS. Twenty four samples corresponded to the low while 178 samples to high blood eosinophil count strata, the latter of which yielded a lower detection rate of bacteria but not viruses. Sixty seven samples were collected from patients with mild bronchiectasis and 135 from patients with moderate‐severe bronchiectasis, the latter of which was associated with a higher detection rate of bacteria, but not viruses. One hundred and eighty one. samples were derived from NSA‐ABOS and 21 from SA‐ABOS. No significant differences in the detection rate of bacteria or viruses were identified. Finally, 57 samples were derived from atopic patients and 120 from non‐atopic patients. Patients in the non‐atopic stratum yielded a higher detection rate of bacteria with or without viruses (*p* = 0.010), but not viruses alone (Figure 3).

## 4 DISCUSSION

Our study has evaluated for the first time, among patients with ABOS, the association between the spectrum of bacteria and/or viruses at steady‐state and AE onset and the clinical characteristics. Bacteria (including PA) were detected fairly common (exceeding 50%), which was associated with greater severity of bronchiectasis. Apart from the variable lung function dynamics, baseline bacterial detection conferred greater exacerbation risks at follow‐up. We also found notable changes in viral but not bacterial detection rate at AE onset compared with steady‐state. Higher blood eosinophil count, moderate‐to‐severe bronchiectasis and non‐atopy were associated with bacterial but not viral detection.

Bacterial infection has been implicated in various CRDs. Both HI and PA are dominant species in bronchiectasis.^21^ Despite the low detection rate,^22^ the outgrowth of *Proteobacteria* (e.g. *Haemophilus* and *Pseudomonas* spp), as detected with 16srRNA sequencing, was more frequently detected in asthmatic patients compared with healthy controls.^15^ Our study has shown a high bacterial detection rate in ABOS, which correlated with bronchiectasis severity. Our findings were consistent with previous reports, which documented that bacterial (esp. PA) detection was associated with greater bronchiectasis severity and more prominent airway inflammation.^23^, ^24^, ^25^, ^26^ Furthermore, a longer duration of symptom onset and higher BSI were associated with bacterial (esp. PA) detection in ABOS, which partly mirrored those of published studies among patients who had predominantly bronchiectasis alone.^26^, ^27^ This implied a notable similarity of bacterial detection between ABOS and bronchiectasis alone.

Among patients with ABOS, bacterial detection was associated with less prominent asthma‐related characteristics, including a lower likelihood of allergic disease family history and current allergic rhinitis, lower blood eosinophil counts, and requiring less corticosteroid treatment. This suggested a counterbalance between atopy and airway infection in bronchiectasis. We have demonstrated a more prominent airflow limitation and a more frequent and accelerated course of AE in patients with bacterial detection. At baseline, patients with ABOS had more frequently received inhaled medications, higher prior exacerbation frequency and more severe airflow limitation. Interestingly, patients with ABOS demonstrated some improvement in airflow limitation if adequately treated during follow‐up. This differed from findings among patients with bronchiectasis alone, who had progressive lung function decline.^28^, ^29^ Therefore, co‐existing asthma and the targeted treatment might be important modifiers of lung function trajectory of ABOS.

Accumulating evidence has unveiled the association between bacteria and/or viruses and AEs, the events with major clinical implications^,^.^14^, ^30^ Previous studies have identified a significant association between viral, but not bacterial, detection and AE onset in patients with bronchiectasis alone.^14^, ^20^, ^31^, ^32^ In ABOS, although both PA and HI were common, they were not directly associated with AE onset. Unlike findings in bronchiectasis alone, we showed no association between the detection of new bacteria and AE onset in ABOS. Similar to bronchiectasis alone, both bacteria (particularly PA) and viruses were significantly associated with ABOS at AE onset. Although caution for data interpretation should be exercised given the small sample sizes, the roles of bacteria cannot be readily extrapolated from bronchiectasis alone.

Despite the minor roles of bacteria at AE onset, viruses played crucial roles at AE onset in ABOS. Our findings of the detection rate and spectrum of viruses mirrored those in bronchiectasis alone.^14^, ^20^, ^32^ A concerning issue is the interaction between bacterial and viral detection. Previous studies have reported greater susceptibility to bacterial infection following viral infection in asthma and bronchiectasis.^14^, ^33^, ^34^, ^35^ However, the association between co‐detection of bacteria and viruses and AE onset has missed the statistical significance, again possibly because of the limited sample sizes.

Some limitations should be considered. The small sample size was a clear limitation and the analysis performed was exploratory. Some patients dropped out because of the long distance from residential places to the study site and the COVID‐19 outbreak. Replication of these correlation analyses and subsequent findings in a larger sample is required with subgroup study analysis considering the dominant taxa of the airway microbiome. We have recruited patients from out‐patient clinics, decreasing the generalizability of our findings to other hospitalized patients. Because of the sufficient sputum yield for routine bacterial culture, we did not enroll patients with asthma alone as a control group herein. We have only included patients with productive cough at baseline, and repeated pathogen detection cannot be performed among all patients. Furthermore, eosinophils were not measured in patients receiving oral corticosteroids and non‐tuberculous mycobacteria culture was not performed. The microbial compositions cannot be addressed by sputum culture alone, and sequencing findings will be reported separately. Because of the lack of existing definitions, AE of ABOS was mainly defined based on the criteria for that of bronchiectasis instead of asthma. However, discriminating the attributable causes of the aggravated symptoms is impractical given the overlapping clinical manifestations of asthma and bronchiectasis.

In summary, bacterial detection at steady‐state correlates with disease severity and future risks of progression in ABOS. Greater attention should be paid to viral detection at AE onset. Our study has highlighted the importance of integrating sputum microbial assessment into clinical practice for ascertaining the dominant pathophysiology (atopy vs. infection) and longitudinal trajectory prediction in ABOS.

## AUTHOR CONTRIBUTIONS

Xiao‐xian Zhang and Jia‐hui He drafted the manuscript. Xiao‐xian Zhang and Wei‐jie Guan contributed to conception and design. Xiao‐xian Zhang and Cui‐xia Pan conducted the experiment. Xiao‐xian Zhang, Jia‐hui He, Cui‐xia Pan, Zhen‐feng He, Hui‐min Li, Zhen‐hong Lin, Xiao‐fen Zhang, Lai‐jian Cen, Ri‐lan Zhang and Ming‐xin Shi were responsible for patient recruitment. Xiao‐xian Zhang, Jia‐hui He and Zhen‐hong Lin collected individual data. Xiao‐xian Zhang and Jia‐hui He performed statistical analyses. Wei‐jie Guan provided a critical review of the manuscript and approved the final submission.

## CONFLICT OF INTEREST STATEMENT

None declared.

## Supporting information

Supporting Information S1

Figure S1

Figure S2

Figure S3

## Data Availability

The data that support the findings of this study are available from the corresponding author upon reasonable request.

## References

[1] Global Initiative for Asthma . Global Strategy for Asthma Management and Prevention; 2023. Accessed October 07, 2022. www.ginasthma.org

[2] Asthma group of Chinese Throacic Society . Guidelines for bronchial asthma prevent and management (2020 edition). Chin J Tubere Respir Dis. 2020;43(12):1023‐1048. 10.3760/cma.j.cn112147-20200618-00721 33333637

[3] Bronchiectasis Expert Consensus Writing Group . Pulmonary infection assembly, Chinese thoracic society. expert consensus on the diagnosis and treatment of adult bronchiectasis in China. Chin J Tubere Respir Dis. 2021;44(4):311‐321. 10.3760/cma.j.cn112147-20200617-00717 33832019

[4] Tiotiu A , Martinez‐Garcia MA , Mendez‐Brea P , Roibas‐Veiga I , Gonzalez‐Barcala FJ . Does asthma‐bronchiectasis overlap syndrome (ABOS) really exist? J Asthma. 2023;60(11):1935‐1941. 10.1080/02770903.2023.2203743 37071539

[5] Xie H , Chen P , Liu L . Analysis of bronchiectasis in hospitalized asthmatic patients: 10‐year experience of a single center. Zhong Hua Yi Xue Za Zhi. 2019;99(16):1210‐1215. 10.3760/cma.j.issn.0376-2491.2019.16.004 31060158

[6] Lan G , Huang C , Liu Y , et al. How does comorbid bronchiectasis affect asthmatic patients? A meta‐analysis. J Asthma. 2021;58(10):1314‐1328. 10.1080/02770903.2020.1784194 32552078

[7] Chan R , Duraikannu C , Lipworth B . Clinical characteristics of the asthma bronchiectasis phenotype. Ann Allergy Asthma Immunol. 2023;130(3):362‐364. 10.1016/j.anai.2022.11.024 36503068

[8] Choi H , Lee H , Ryu J , et al. Bronchiectasis and increased mortality in patients with corticosteroid‐dependent severe asthma: a nationwide population study. Ther Adv Respir Dis. 2020;14:1022269226. 10.1177/1753466620963030 PMC7580190 33059535

[9] Kim NY , Lee CH , Jin KN , et al. Clinical deterioration and lung function change in patients with concomitant asthma and bronchiectasis. J Allergy Clin Immunol Pract. 2022;10(10):2607‐2613. 10.1016/j.jaip.2022.05.026 35690367

[10] Mao B , Yang JW , Lu HW , Xu JF . Asthma and bronchiectasis exacerbation. Eur Respir J. 2016;47(6):1680‐1686. 10.1183/13993003.01862-2015 27076584

[11] Ferri S , Crimi C , Campisi R , et al. Impact of asthma on bronchiectasis severity and risk of exacerbations. J Asthma. 2022;59(3):469‐475. 10.1080/02770903.2020.1857395 33256490

[12] Aliberti S , Goeminne PC , O'Donnell AE , et al. Criteria and definitions for the radiological and clinical diagnosis of bronchiectasis in adults for use in clinical trials: international consensus recommendations. Lancet Respir Med. 2022;10(3):298‐306. 10.1016/s2213-2600(21)00277-0 34570994

[13] Hill AT , Haworth CS , Aliberti S , et al. Pulmonary exacerbation in adults with bronchiectasis: a consensus definition for clinical research. Eur Respir J. 2017;49(6):1700051. 10.1183/13993003.00051-2017 28596426

[14] Chen CL , Huang Y , Yuan JJ , et al. The roles of bacteria and viruses in bronchiectasis exacerbation: a prospective study. Arch Bronconeumol. 2020;56(10):621‐629. 10.1016/j.arbres.2019.12.010 33994634

[15] Huang YJ , Nelson CE , Brodie EL , et al. Airway microbiota and bronchial hyperresponsiveness in patients with suboptimally controlled asthma. J Allergy Clin Immunol. 2011;127(2):372‐381. 10.1016/j.jaci.2010.10.048 21194740 PMC3037020

[16] Huang YJ , Nariya S , Harris JM , et al. The airway microbiome in patients with severe asthma: associations with disease features and severity. J Allergy Clin Immunol. 2015;136(4):874‐884. 10.1016/j.jaci.2015.05.044 26220531 PMC4600429

[17] Chalmers JD , Goeminne P , Aliberti S , et al. The bronchiectasis severity index. An international derivation and validation study. Am J Respir Crit Care Med. 2014;189(5):576‐585. 10.1164/rccm.201309-1575oc 24328736 PMC3977711

[18] Guan WJ , Yuan JJ , Li HM , et al. Proteobacteria community compositions correlate with bronchiectasis severity. Int J Tubercul Lung Dis. 2018;22(9):1095‐1105. 10.5588/ijtld.18.0037 30092878

[19] Martinez‐Garcia MA , Athanazio RA , Girón R , et al. Predicting high risk of exacerbations in bronchiectasis: the E‐FACED score. Int J Chronic Obstr Pulm Dis. 2017;12:275‐284. 10.2147/copd.s121943 PMC5279836 28182132

[20] Gao YH , Guan WJ , Xu G , et al. The role of viral infection in pulmonary exacerbations of bronchiectasis in adults: a prospective study. Chest. 2015;147(6):1635‐1643. 10.1378/chest.14-1961 25412225 PMC7094490

[21] Guan WJ , Gao YH , Xu G , et al. Sputum bacteriology in steady‐state bronchiectasis in Guangzhou, China. Int J Tubercul Lung Dis. 2015;19(5):610‐619. 10.5588/ijtld.14.0613 25868032

[22] Normansell R , Sayer B , Waterson S , Dennett EJ , Del Forno M , Dunleavy A . Antibiotics for exacerbations of asthma. Cochrane Database Syst Rev. 2018;6(6):D2741. 10.1002/14651858.cd002741.pub2 PMC6513273 29938789

[23] Wang R , Ding S , Lei C , Yang D , Luo H . The contribution of Pseudomonas aeruginosa infection to clinical outcomes in bronchiectasis: a prospective cohort study. Ann Med. 2021;53(1):459‐469. 10.1080/07853890.2021.1900594 33754900 PMC7993380

[24] Figueiredo MR , Lomonaco I , Araújo AS , Lundgren F , Pereira EDB . Isolation of and risk factors for airway infection with Pseudomonas aeruginosa in patients with non‐cystic fibrosis bronchiectasis. J Bras Pneumol. 2021;47(3):e20210017. 10.36416/1806-3756/e20210017 34190862 PMC8332711

[25] Guan WJ , Gao YH , Xu G , et al. Characterization of lung function impairment in adults with bronchiectasis. PLoS One. 2014;9(11):e113373. 10.1371/journal.pone.0113373 25405614 PMC4236163

[26] Guan WJ , Gao YH , Xu G , et al. Effect of airway Pseudomonas aeruginosa isolation and infection on steady‐state bronchiectasis in Guangzhou, China. J Thorac Dis. 2015;7(4):625‐636. 25973228 10.3978/j.issn.2072-1439.2015.04.04 PMC4419315

[27] Kwok WC , Ho JCM , Tam TCC , Ip MSM , Lam DCL . Risk factors for Pseudomonas aeruginosa colonization in non‐cystic fibrosis bronchiectasis and clinical implications. Respir Res. 2021;22(1):132. 10.1186/s12931-021-01729-5 33910573 PMC8080398

[28] Martinez‐Garcia MA , Oscullo G , Posadas T , et al. Pseudomonas aeruginosa and lung function decline in patients with bronchiectasis. Clin Microbiol Infect. 2021;27(3):428‐434. 10.1016/j.cmi.2020.04.007 32311472

[29] Martinez‐García MA , Oscullo G , Posadas T , et al. Factors associated with lung function decline in adult patients with stable non‐cystic fibrosis bronchiectasis. Chest. 2007;132(5):1565‐1572. 10.1378/chest.07-0490 17998359

[30] Wedzicha JA , Seemungal TA . COPD exacerbations: defining their cause and prevention. Lancet. 2007;370(9589):786‐796. 10.1016/s0140-6736(07)61382-8 17765528 PMC7134993

[31] Guan WJ , Yuan JJ , Li HM , et al. Altered community compositions of Proteobacteria in adults with bronchiectasis. Int J Chronic Obstr Pulm Dis. 2018;13:2173‐2182. 10.2147/copd.s159335 PMC6054765 30140149

[32] Huang Y , Chen CL , Cen LJ , et al. Sputum pathogen spectrum and clinical outcomes of upper respiratory tract infection in bronchiectasis exacerbation: a prospective cohort study. Emerg Microb Infect. 2023;12(1):2202277. 10.1080/22221751.2023.2202277 PMC10167879 37038356

[33] Kloepfer KM , Lee WM , Pappas TE , et al. Detection of pathogenic bacteria during rhinovirus infection is associated with increased respiratory symptoms and asthma exacerbations. J Allergy Clin Immunol. 2014;133(5):1301‐1307. 10.1016/j.jaci.2014.02.030 24698319 PMC4047978

[34] George SN , Garcha DS , Mackay AJ , et al. Human rhinovirus infection during naturally occurring COPD exacerbations. Eur Respir J. 2014;44(1):87‐96. 10.1183/09031936.00223113 24627537

[35] Rynda‐Apple A , Robinson KM , Alcorn JF . Influenza and bacterial superinfection: illuminating the immunologic mechanisms of disease. Infect Immun. 2015;83(10):3764‐3770. 10.1128/iai.00298-15 26216421 PMC4567631

