# Supplementary material for: Bacteria and viruses and clinical outcomes of asthma‐bronchiectasis overlap syndrome: A cohort study
Source: Clin Transl Allergy. 2024 Jan 11;14(1):e12331. doi: 10.1002/clt2.12331 (PMC10784706; doi:10.1002/clt2.12331)
Supplement: Supplementary file 1 — Supporting Information S1 [file CLT2-14-e12331-s003.doc]

**Online supplement**

**Bacteria and Viruses and clinical outcomes of Asthma-Bronchiectasis Overlap Syndrome : A Cohort Study**

Xiao-xian Zhang ^a#^, Ph.D., Jia-hui He ^a#^, M. MSc., Cui-xia Pan ^a#^, Ph.D., Zhen-feng He ^a^, Ph.D., Hui-min Li ^a^, M.T., Zhen-hong Lin ^a^ , M. MSc., Xiao-fen Zhang ^a^, M.T., Lai-jian Cen ^a^, Ph.D., Ri-lan Zhang ^a^, M. MSc., Ming-xin Shi ^a^, M. MSc., Wei-jie Guan ^a,b,c*^ , M. D.

^a^ State Key Laboratory of Respiratory Disease, National Clinical Research Center for Respiratory Disease, Guangzhou Institute for Respiratory Health, The First Affiliated Hospital of Guangzhou Medical University, Guangzhou, Guangdong, China

**^#^ Drs. Xiao-xian Zhang, Jia-hui He and Cui-xia Pan contributed equally to the work**

*** Wei-jie Guan contributed to the work and was the lead contact**

**Corresponding author : Wei-jie Guan, M. D., State Key Laboratory of Respiratory Disease, National Clinical Research Center for Respiratory Disease, Guangzhou Institute for Respiratory Health, The First Affiliated Hospital of Guangzhou Medical University, Address: 28 Qiaozhong Road Middle, Guangzhou, Guangdong, China, E-mail: battery203@163.com**

**eTable 1. Baseline clinical characteristics in patients with ABOS with or without viral detection when clinically stable**

|  | **Virus**  **detected (n=14)** | **No virus**  **detected (n=57)** | ***P value*** |
| --- | --- | --- | --- |
| Age (yrs) | 46.3±10.2 | 49.2±13.7 | 0.462 |
| Females, n(%) | 11.0 (78.6%) | 35.0 (61.4%) | 0.351 |
| Body-mass index (kg/m²) | 21.5 (5.2) | 21.4 (4.4) | 0.639 |
| Duration of symptom onset (yrs) | 22.5 (20.5) | 17.0 (15.0) | 0.351 |
| Wheeze, n(%) | 5.0 (35.7%) | 21.0 (36.8%) | >0.999 |
| 24-hr sputum **≥**10ml, n(%) | 9.0 (64.3%) | 37.0 (64.9%) | >0.999 |
| Previous smoker, n(%) | 0.0 (0.0%) | 5.0 (8.8%) | 0.575 |
| Family history of allergic disease, n(%) | 7.0(50.0%) | 22.0 (38.6%) | 0.547 |
| Rhinitis or sinusitis, n(%) | 9.0 (64.3%) | 42.0 (73.7%) | 0.517 |
| Allergic rhinitis, n(%) | 6.0 (42.9%) | 30.0 (52.6%) | 0.563 |
| Influenza vaccination within 1 year, n(%) | 3.0 (21.4%) | 5.0 (8.8%) | 0.342 |
| Pneumococcal vaccination within 5 years, n(%) | 3.0 (21.4%) | 7.0 (12.3%) | 0.402 |
| Exacerbations frequency in the previous year | 1.0 (2.3) | 1.0 (2.0) | 0.856 |
| No. of patients hospitalized in the previous year | 0.0 (1.0) | 0.0 (0.0) | 0.452 |
| Severe asthma, n(%) | 3.0 (21.4%) | 7.0 (12.3%) | 0.402 |
| Bronchiectasis Severity Index | 4.5 (6.5) | 5.0 (5.0) | 0.948 |
| E-FACED score | 2.5 (2.3) | 2.0 (2.0) | 0.888 |
| HRCT Reiff score | 7.6±4.2 | 8.8±4.1 | 0.355 |
| No. of bronchiectatic lobes | 3.5 (3.0) | 4.0 (2.0) | 0.200 |
| FEV_1_%pred(%) | 57.5±16.4 | 56.8±18.4 | 0.891 |
| FEV_1_/FVC(%) | 61.3±10.6 | 60.2±12.9 | 0.774 |
| White blood cells (*10^9/L) | 7.0±1.6 | 7.3±2.0 | 0.546 |
| Blood neutrophils (%) | 61.7±8.4 | 61.9±9.3 | 0.944 |
| Blood eosinophils (%) | 1.8 (3.0) | 2.2 (2.2) | 0.983 |
| C-reactive protein # (mg/dL) | 0.4 (0.4) | 0.3 (0.6) | 0.864 |
| Total IgE# (KU/ml) | 135.0 (246.6) | 53.0 (140.9) | 0.241 |
| FeNO# (ppb) | 13.5 (10.8) | 15.0 (20.0) | 0.576 |
| Sputum neutrophils # (%) | 95.3 (6.6) | 96.8 (6.3) | 0.897 |
| Sputum eosinophils # (%) | 1.9 (4.4) | 1.0 (1.4) | 0.095 |
| Low-dose macrolides, n(%) | 2.0 (14.3%) | 8.0 (14.0%) | >0.999 |
| ICS, n(%) | 5.0 (35.7%) | 19.0 (33.3%) | >0.999 |
| OCS, n(%) | 0.0 (0.0%) | 2.0 (3.5%) | >0.999 |
| LAMA, n(%) | 6.0 (42.9%) | 27.0 (47.7%) | 0.776 |
| LABA, n(%) | 5.0 (35.7%) | 26.0 (45.6%) | 0.561 |

Abbreviation: ABOS = Asthma-Bronchiectasis Overlap Syndrome; FEV1%pred = the percentage of predicted forced expiratory volume in one second; FEV_1_/FVC = Forced expiratory volume in one second / Forced vital capacity; IgE = Immunoglobulin E; FeNO = Fractional exhaled nitric oxide; ICS = Inhaled corticosteroids; OCS = Oral corticosteroids; LAMA = Long-acting muscarinic antagonists; LABA = Long-acting Beta-agonists.

# 68 patients had undergone sputum cytology examination, 65 patients had undergone total IgE testing, 51 patients had undergone FeNO testing, 67 patients had undergone C-reactive protein testing.

Bold font: P<0.05, statistically significant differences.

**eTable 2. Baseline characteristics of patients with ABOS and bronchiectasis alone.**

|  | **ABOS**  **(n=81)** | **Bronchiectasis alone (n=107)** | ***P*** |
| --- | --- | --- | --- |
| Age (yrs) | 48.4 (21.3) | 47.4 (20.7) | 0.742 |
| Females, n(%) | 54.0 (66.7%) | 60.0 (56.1%) | 0.141 |
| Body-mass index (kg/m²) | 21.4 (4.6) | 20.1 (4.5) | **0.008** |
| Duration of symptom onset (yrs) | 17.0 (15.5) | 16.3 (19.1) | 0.699 |
| Previous smoker, n(%) | 7.0 (8.6%) | 7.0 (6.5%) | 0.587 |
| Influenza vaccination within 1 year, n(%) | 8.0 (9.9%) | 0.0 (0.0%) | **0.001** |
| Pneumococcal vaccination within 5 years, n(%) | 10.0 (12.3%) | 0.0 (0.0%) | **<0.001** |
| Exacerbations frequency in the previous year | 1.0 (2.0) | 1.0 (1.0) | **0.029** |
| Severe exacerbations frequency in the previous 2 years | 0.0 (0.0) | 0.0 (1.0) | 0.231 |
| Bronchiectasis Severity Index | 5.0 (5.0) | 6.0 (6.0) | 0.849 |
| No. of bronchiectatic lobes | 4.0 (2.0) | 4.0 (2.0) | 0.886 |
| FEV_1_ pred (%) | 57.7 (29.0) | 67.5 (37.3) | **0.009** |
| Low-dose macrolides, n(%) | 11.0 (13.6%) | 18.0 (16.8%) | 0.542 |
| ICS (No.,%) | 30.0 (37.0%) | 11.0 (10.3%) | **<0.001** |
| LAMA (No.,%) | 36.0 (44.4%) | 26.0 (24.3%) | **0.004** |
| LABA (No.,%) | 37.0 (45.7%) | 14.0 (13.1%) | **<0.001** |

Abbreviation：ABOS: Asthma-Bronchiectasis Overlap Syndrome; FEV1%pred: the percentage of predicted forced expiratory volume in one second; FEV_1_/FVC: the ratio of forced expiratory volume in one second / forced vital capacity; ICS: Inhaled corticosteroids; LAMA: Long-acting muscarinic antagonists; LABA: Long-acting Beta-agonists.

Bold font: P<0.05, statistically significant differences.

**eTable 3. Baseline characteristics of patients with ABOS in the whole cohort and AE cohort.**

|  | **ABOS in**  **the whole cohort**  **(n=75)** | **ABOS in**  **AE cohort**  **(n=27)** | ***P*** |
| --- | --- | --- | --- |
| Age (yrs) | 48.7±12.8 | 48.1±13.4 | 0.837 |
| Females, n(%) | 50.0 (66.7%) | 19.0 (70.4%) | 0.724 |
| Body-mass index (kg/m²) | 21.5 (4.7) | 21.4 (5.7) | 0.764 |
| Duration of symptom onset (yrs) | 17.0 (15.0) | 20.0 (11.0) | 0.404 |
| Previous smoker, n(%) | 5.0 (6.7%) | 0.0 (0.0%) | 0.321 |
| Influenza vaccination within 1 year, n(%) | 8.0 (10.7%) | 0.0 (0.0%) | 0.106 |
| Pneumococcal vaccination within 5 years, n(%) | 10.0 (13.3%) | 4.0 (14.8%) | >0.999 |
| Exacerbations frequency in the previous year | 1.0 (2.0) | 1.0 (2.0) | 0.217 |
| No. of patients hospitalized in the previous year | 0.0 (0.0) | 0.0 (1.0) | 0.621 |
| Severe asthma, n(%) | 12.0 (16.0%) | 2.0 (7.4%) | 0.344 |
| Bronchiectasis Severity Index | 5.0 (4.0) | 6.0 (4.0) | 0.378 |
| E-FACED score | 2.0 (2.0) | 3.0 (3.0) | 0.446 |
| HRCT Reiff score | 8.0 (7.0) | 8.0 (5.0) | 0.247 |
| The No. of bronchiectatic lobes | 4.0 (2.0) | 5.0 (3.0) | 0.138 |
| FEV_1_ pred (%) | 57.3±18.3 | 53.9±18.7 | 0.403 |
| FEV_1_/FVC (%) | 60.8±12.6 | 70.0±12.8 | 0.965 |
| White blood cells (*10^9/L) | 7.3±1.9 | 7.0±1.9 | 0.568 |
| Blood neutrophils (%) | 4.3 (2.2) | 4.4 (2.9) | 0.838 |
| Blood eosinophils (%) | 0.2 (0.1) | 0.1 (0.1) | 0.264 |
| C-reactive protein # (mg/dL) | 0.3 (0.5) | 0.5 (0.9) | 0.227 |
| Low-dose macrolides, n(%) | 11.0 (14.7%) | 5.0 (18.5%) | 0.637 |
| ICS, n(%) | 26.0 (34.7%) | 10.0 (37.0%) | 0.825 |
| OCS, n(%) | 3.0 (4.0%) | 1.0 (3.7%) | >0.999 |
| LAMA, n(%) | 34.0 (45.3%) | 13.0 (48.1%) | 0.801 |
| LABA, n(%) | 33.0 (44.0%) | 13.0 (48.1%) | 0.710 |

Abbreviation: ABOS: Asthma-Bronchiectasis Overlap Syndrome; FEV1%pred: the percentage of predicted forced expiratory volume in one second; FEV_1_/FVC: the ratio of forced expiratory volume in one second / forced vital capacity; ICS: Inhaled corticosteroids; OCS: Oral corticosteroids; LAMA=Long-acting muscarinic antagonists; LABA: Long-acting Beta-agonists.

# 71 patients had CRP examination.

Bold font：P＜0.05, the difference was statistically significant.

**eTable 4. Baseline characteristics of the whole cohort and AE cohort among patients with bronchiectasis alone**

|  | **The whole cohort (n=107)** | **AE cohort (n=33)** | ***P value*** |
| --- | --- | --- | --- |
| Age (yrs) | 47.4 (20.7) | 54.1 (22.3) | 0.238 |
| Females, n(%) | 60.0 (56.1%) | 19.0 (57.6%) | 0.879 |
| Body-mass index (kg/m²) | 20.4±2.9 | 20.1±2.6 | 0.536 |
| Duration of symptom onset (yrs) | 16.3 (19.1) | 18.2 (16.4) | 0.833 |
| Previous smoker, n(%) | 7.0 (6.5%) | 4.0 (12.1%) | 0.287 |
| Exacerbations frequency in the previous year | 1.0 (1.0) | 2.0 (1.0) | 0.025 |
| Severe exacerbations frequency in the previous 2 years | 0.0 (1.0) | 0.0 (1.0) | 0.931 |
| Bronchiectasis Severity Index | 6.0 (6.0) | 8.0 (6.0) | 0.067 |
| The No. of bronchiectatic lobes | 4.0 (2.0) | 4.0 (2.0) | 0.081 |
| FEV_1_ pred (%) | 67.5 (37.3) | 56.3 (39.5) | 0.238 |
| Low-dose macrolides, n(%) | 18.0 (16.8%) | 4.0 (12.1%) | 0.597 |
| ICS, n(%) | 11.0 (10.3%) | 4.0 (12.1%) | 0.753 |
| OCS, n(%) | 26.0 (24.3%) | 13.0 (39.4%) | 0.091 |
| LAMA, n(%) | 14.0 (13.1%) | 3.0 (9.1%) | 0.762 |

Abbreviation: Bx: bronchiectasis alone; FEV_1_%pred: the percentage of predicted forced expiratory volume in one second; ICS: Inhaled corticosteroids; OCS: Oral corticosteroids; LAMA: Long-acting muscarinic antagonists.

**eTable 5. Symptoms of AEs With Different Virus Detection status among patients with ABOS**

|  | **Virus (+)**  **(n=11)** | **Virus (-) (n=27)** | ***P*** | **V+B-**  **(n=6)** | **Others**  **(n=32)** | ***P*** |
| --- | --- | --- | --- | --- | --- | --- |
| Fever and or shivery, n(%) | 3.0 (27.3%) | 6.0 (22.2%) | 1.000 | 2.0 (33.3%) | 7.0 (21.9%) | 0.613 |
| Headache, n(%) | 3.0 (27.3%) | 6.0 (22.2%) | 1.000 | 0.0 (0.0%) | 9.0 (28.1%) | 0.303 |
| Ocular itching, n(%) | 3.0 (27.3%) | 4.0 (14.8%) | 0.390 | 1.0 (16.7%) | 6.0 (18.8%) | 1.000 |
| Other systemic pain, n(%) | 1.0 (9.1%) | 1.0 (3.7%) | 0.501 | 0.0 (0.0%) | 2.0 (6.3%) | 1.000 |
| Runny nose, n(%) | 6.0 (54.5%) | 7.0 (25.9%) | 0.092 | 3.0 (50.0%) | 10.0 (31.3%) | 0.392 |
| Blocked or stuffy nose, n(%) | 3.0 (27.3%) | 7.0 (25.9%) | 1.000 | 1.0 (16.7%) | 9.0 (28.1%) | 1.000 |
| Sneezing, n(%) | 3.0 (27.3%) | 4.0 (14.8%) | 0.390 | 1.0 (16.7%) | 6.0 (18.8%) | 1.000 |
| Sore throat, n(%) | 8.0 (72.2%) | 12.0 (44.4%) | 0.160 | 4.0 (66.7%) | 16.0 (50.0%) | 0.663 |
| Hoarseness, n(%) | 1.0 (9.1%) | 2.0 (7.4%) | 1.000 | 3.0 (9.4%) | 0.0 (0.0%) | 1.000 |
| Increased cough frequency, n(%) | 10.0 (90.9%) | 24.0 (88.9%) | 1.000 | 5.0 (83.3%) | 29.0 (90.6%) | 0.513 |
| Increased sputum volume, n(%) | 10.0 (90.9%) | 25.0 (92.6%) | 1.000 | 5.0 (83.3%) | 30.0 (93.8%) | 0.412 |
| Increased sputum purulence, n(%) | 9.0 (81.8%) | 20.0 (74.1%) | 1.000 | 5.0 (83.3%) | 24.0 (75.0%) | 1.000 |
| Aggravated breathlessness, n(%) | 9.0 (81.8%) | 15.0 (55.6%) | 0.160 | 4.0 (66.7%) | 20.0 (62.5%) | 1.000 |
| Fatigue/malaise, n(%) | 6.0 (54.4%) | 17.0 (63.0%) | 0.630 | 2.0 (33.3%) | 21.0 (65.6%) | 0.188 |
| Hemoptysis, n(%) | 0.0 (0.0%) | 3.0 (11.1%) | 0.542 | 0.0 (0.0%) | 3.0 (9.4%) | 1.000 |

Notes: AE: acute exacerbations of bronchiectasis; Virus (+): any viruses detected; Virus (-): no viruses detected; V+B−: viruses detected but no pathogenic bacteria detected; Others: including B−V−, B+V−, B+V+;

B−V−: no bacteria and viruses detected; B+V−: any pathogenic bacteria detected but no viruses detected; B+V+: both bacteria and viruses detected.

Data are presented as n (%).

Kruskal-Wallis comparison with Bonferroni correction was applied.

**Figure legends**

**eFigure 1 Lung function variation during follow-up in patients with ABOS**

1. FEV_1_ pred% at baseline compared with follow-up in patients with ABOS;
2. The improvement of FEV_1_pred% from baseline to follow-up in bacterial culture positive group compared with that in bacterial culture negative group;
3. The improvement of FEV_1_pred% from baseline to follow-up in *Pseudomonas aeruginosa* (*PA*) group compared with that in the non-*PA* group;
4. The improvement of FEV_1_pred% from baseline to follow-up in viral detection positive group compared with that in viral detection negative group.

Abbreviation: ABOS: Asthma-Bronchiectasis Overlap Syndrome, FEV_1_%pred: the percentage of predicted forced expiratory volume in one second; *PA*: *Pseudomonas aeruginosa*.

**eFigure 2 The severity of lower airway symptoms assessed with the visual analog scale in patients with ABOS**

1. (c) The VAS score during AEs. AE symptoms including fatigue/malaise, wheeze, breathlessness, sputum purulence, sputum volume, cough. (b)(d)The change in VAS score between AEs and stable visits.

Notes: AE: acute exacerbation of bronchiectasis; VAS: visual analog scale; delta VAS score: The VAS score at AE visit was subtracted from VAS score at stead-state visit. Any virus denotes any virus being detected. No virus denotes viral detection negative. V−B+: any pathogenic bacteria being detected without viruses being detected. Others: including no bacteria and viruses detected, any pathogenic bacteria detected but no viruses detected, both bacteria and viruses detected.

**eFigure 3 The systemic inflammations across different groups**

Notes: AE: acute exacerbation of bronchiectasis. Any virus denotes any virus being detected. No virus denotes viral detection negative.

1. The difference in white blood cell count and C-reactive protein during AEs between Any virus group and No virus group, and between AE visits and steady-state visits; (B) The difference in inflammatory cell percentage during AEs between Any virus group and No virus group, and between AE visits and steady-state visits; (C) The difference in inflammatory cell count during AEs between Any virus group and No virus group, and between AE visits and steady-state visits.
