# Supplementary figures and images for: Bacteria and viruses and clinical outcomes of asthma‐bronchiectasis overlap syndrome: A cohort study
Source: Clin Transl Allergy. 2024 Jan 11;14(1):e12331. doi: 10.1002/clt2.12331 (PMC10784706; doi:10.1002/clt2.12331)

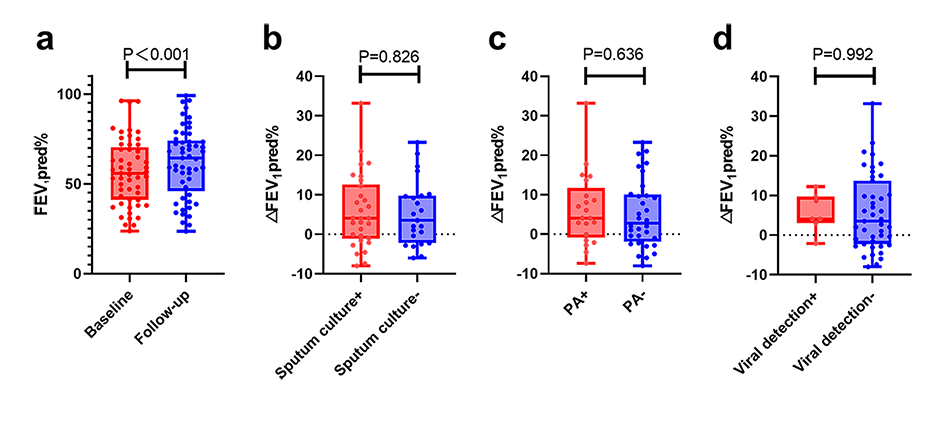

Supplement: Supplementary file 2 — Figure S1 [file CLT2-14-e12331-s001.tif]

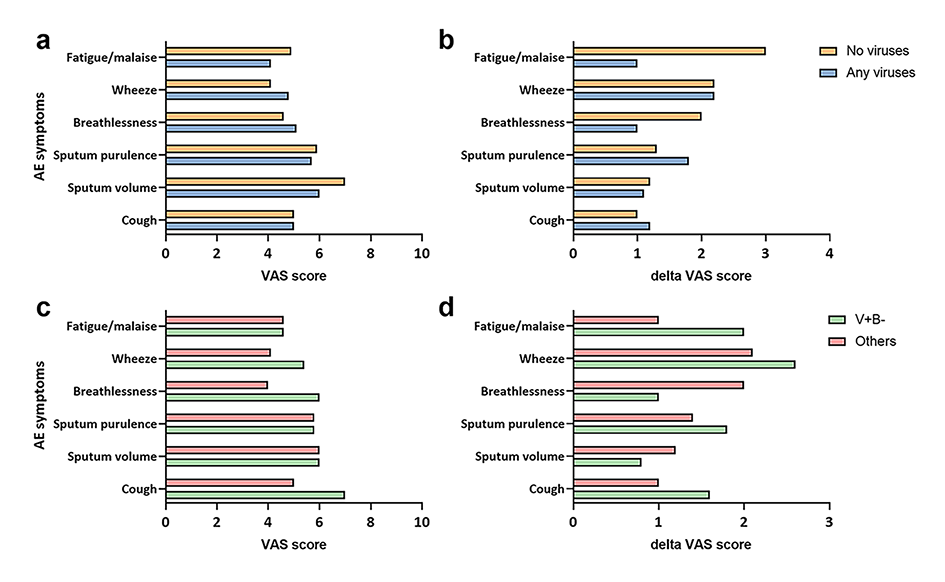

Supplement: Supplementary file 3 — Figure S2 [file CLT2-14-e12331-s002.tif]

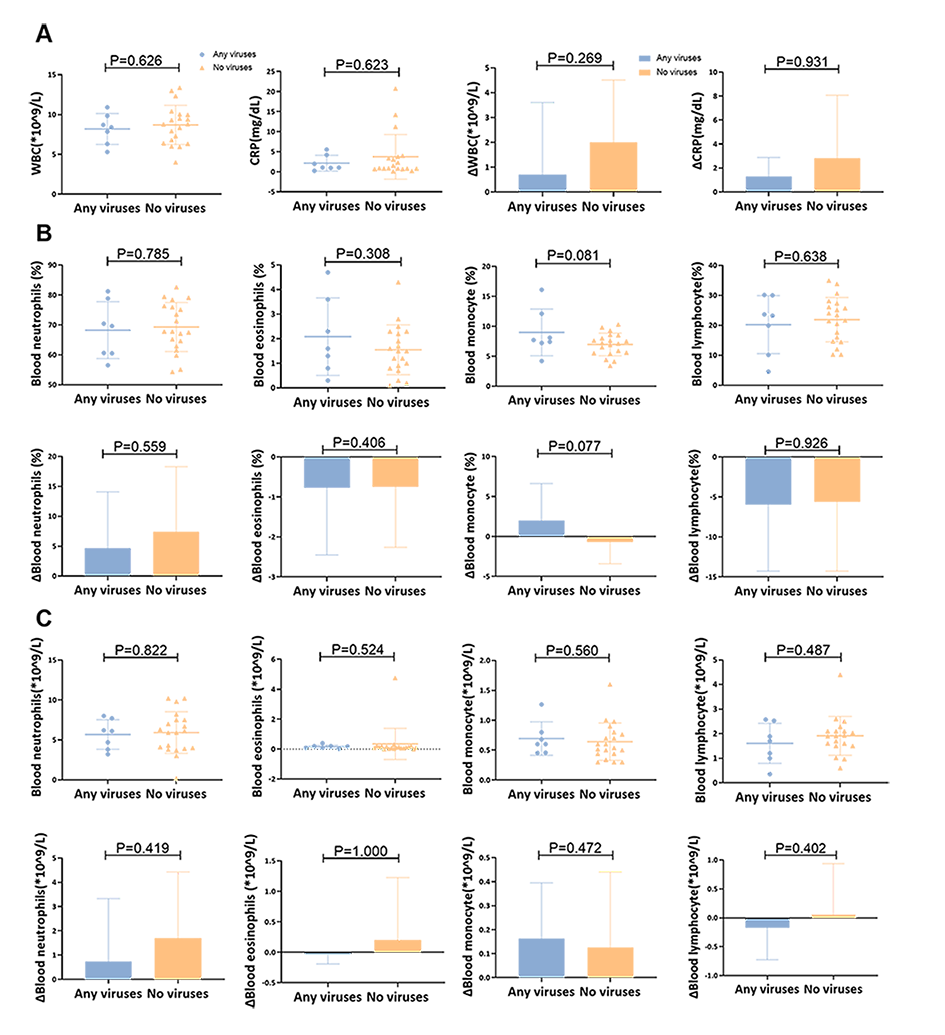

Supplement: Supplementary file 4 — Figure S3 [file CLT2-14-e12331-s004.tif]
